# Implementation of and Experimentation with Ground-Penetrating Radar for Real-Time Automatic Detection of Buried Improvised Explosive Devices

**DOI:** 10.3390/s22228710

**Published:** 2022-11-11

**Authors:** Pachara Srimuk, Akkarat Boonpoonga, Kamol Kaemarungsi, Krit Athikulwongse, Sitthichai Dentri

**Affiliations:** 1 Research Center of Innovation Digital and Electromagnetic Technology, Electrical Engineering, Department of Electrical and Computer Engineering, Faculty of Engineering, King Mongkut’s University of Technology North Bangkok, Bangkok 10800, Thailand; 2 National Electronics and Computer Technology Center, National Science and Technology Development Agency, Pathumthani 12120, Thailand; 3 Department of Electronics Engineering Technology, Collage of Industrial Technology, King Mongkut’s University of Technology North Bangkok, Bangkok 10800, Thailand

**Keywords:** ground-penetrating radar, GPR, region-based convolutional neural network, R-CNN, improvised explosive devices, IED, automatic detection, ultra-wideband antenna, UWB antenna

## Abstract

This paper proposes the implementation of and experimentation with GPR for real-time automatic detection of buried IEDs. GPR, consisting of hardware and software, was implemented. A UWB antenna was designed and implemented, particularly for the operation of the GPR. The experiments were conducted in order to demonstrate the real-time automatic detection of buried IEDs using GPR with an R-CNN algorithm. In the experiments, the GPR was mounted on a pickup truck and a maintenance train in order to find the IEDs buried under a road and a railway, respectively. B-scan images were collected using the implemented GPR. R-CNN-based detection for the hyperbolic pattern, which indicates the buried IED, was performed along with pre-processing, for example, using zero offset removal, and background removal and filtering. Experimental results in terms of detecting the hyperbolic pattern in B-scan images were shown and verified that the proposed GPR system is superior to the conventional one using region analysis processing-based detection. Results also showed that pre-processing is required in order to improve and/or clean the hyperbolic pattern before detection. The GPR can automatically detect IEDs buried under roads and railways in real time by detecting the hyperbolic pattern appearing in the collected B-scan image.

## 1. Introduction

In the last two decades, there has been an increase in the number of terrorist attacks, especially in Southern Thailand. One of the occurrences happening most often in terrorist attacks is explosions on people’s transportation, including on roads and trains [1,2]. An improvised explosive device (IED) made of urea fertilizer and diesel packed with a gas tank was buried under a road and railway. The visual inspection of the buried IED is somewhat difficult and dangerous for soldier patrols. A technology that can find and detect the buried IED is therefore required. Ground-penetrating radar (GPR) is one of the most popular methods that can resolve the underlying problem. It has been of considerable interest for research activities in military operations, especially for exploring the landmines or IEDs buried underground [3,4,5]. GPR, which is a geophysical non-destructive method that is based on the basic principle of backscattered electromagnetic (EM) waves, can image the subsurface. GPR can be divided into two main technologies, namely stepped-frequency continuous wave (SFCW) and pulse radar [6,7,8,9]. The first transmits continuous signals whose frequency is stepped, propagated underground, and then backscattering signals are received. The received scattering signals, including phase and magnitude responses, are transformed to time-domain signals interpreted as underground objects. The main advantages of the SFCW-based radar are its good range resolution, high signal-to-noise ratio, and cost-effective solution. However, the RF and digital circuitries are complicated. The second technology transmits short EM pulses that are propagated underground and then receive backscattering pulses as well. The main advantages of the pulse-based radar are its simple hardware design and signal processing. However, high-power transmission and a high-speed analog-to-digital converter (ADC) are required. One can choose SFCW- or pulse-based GPR technologies to find buried IEDs and landmines made of metallic and non-metallic materials.

GPR has been widely used to detect landmines throughout the world because it allows patrols to pinpoint the location of landmines without disturbing the ground [3,4,5]. In [10], a NIITEK-manufactured GPR was mounted on a vehicle to survey three geographically different places. The data collected by the GPR were processed using a context-dependent framework for landmine detection and discrimination. A technique using feature-based rules, adaptive whitening, and order statistics has also been developed along with the NIITEK GPR for landmine detection [11]. The NIITEK GPR employed in [10,11,12,13,14,15,16,17] is a wideband impulse radar. However, since the NIITEK GPR is a commercial product developed for general purposes, performing advance signal processing tasks can only carried out offline. That is, the GPR data must be completely collected and subsequently processed by using signal processing techniques. The development of a GPR operated along with signal processing to achieve the real-time automatic detection of buried objects, including IEDs and landmines, is therefore required for field operations.

In order to achieve real-time automatic detection of buried IEDs, there is a need for an efficient detection technique along with GPR to be implemented specifically for real-time operations. Currently, we have proposed a method of automatic detection for buried IEDs using GPR [1]. Detection was achieved using complex natural frequencies extracted by using the short-time matrix pencil method (STMPM). However, only simulations were conducted to illustrate the performance of the proposed method. Another technique that can be used to detect landmines is based on the pattern recognitions of the GPR images, in which the hyperbolic pattern is associated with buried objects [18,19,20,21,22,23,24]. In [25,26,27,28,29], an image analysis technique based on the modified Hough transform was proposed to localize objects by finding straight lines and hyperbolic arcs in the GPR images. In [27], a fuzzy clustering approach was proposed to recognize hyperbolic signatures in the subsurface GPR image. An algorithm detecting the apex of the hyperbolic patterns by fitting the analytical function of the hyperbolic signature to the profile edge dots detected with a filter was proposed [22]. In this approach, the hyperbola’s frequent misshapenness due to the operation of GPR in the field was resolved.

One of the most popular candidates for the image detection technique employed to localize objects within an image is the region-based convolutional neural network (R-CNN). The R-CNN was first proposed in [30] by applying the convolution neural network (CNN) to object detection and introducing the use of a selective search algorithm to create a group of region proposals for each input image, and then using a CNN to extract their features and to train them for the support vector machine-based (SVM) classification. The R-CNN has been widely used in many applications and modified to improve its accuracy, efficiency, and computational speed as seen in [31,32,33,34,35]. However, there is no research on applying the R-CNN to the real-time automatic detection of buried IEDs by using GPR.

In addition to signal processing for the detection of buried objects, another essential component of the GPR implementation is an ultra-wideband (UWB) antenna, whose response should cover a wide bandwidth. The radiation pattern of the antenna used for GPR should be unidirectional with a high gain characteristic. A bowtie antenna with two folded arms at 20-degree angles was designed with a hollowed cavity that achieved |*S*_11_| below −10 dB covering the frequency of 250 MHz to 850 MHz. The maximum gain at 850 MHz was 4 dBi with the echo amplitude of less than 0.1 V [36]. In addition, the bowtie antenna with folded arms of 30 degrees with reflector-backed folded structures, which achieved |*S*_11_| < −10 dB along the frequency of 0.5 GHz to 3 GHz, had the maximum gain of 12 dBi [37]. The simple antenna design with a low-profile structure of a TEM horn consisting of a single arm connected to the ground geometry was proposed to be operated at 140 MHz to 510 MHz with the gain of 4.9 dBi and to radiate a unidirectional pattern [38]. The antipodal tapered slot antenna designed with artificial material for gain enhancement with |*S*_11_| < −10 dB, covering 2 GHz to 7 GHz, can achieve 2 dB of gain, higher than that of the previously proposed antenna [39]. Epoxy-glass substrate was applied to design the tapered antenna with two arms and integrated with different layers, referred to as a double-side tapered antenna, whose operating frequency can cover 5.5 GHz to 10.5 GHz with the time-domain characteristic of reflection at the time of 3.85 ns [40]. The main contributions of this paper are summarized as follows. First, an ultra-wideband (UWB) scutcheon antenna was designed and implemented, particularly for IED detection using the GPR system. Second, the hardware and software of the GPR system were implemented in order to achieve the real-time automatic detection of IEDs buried under roads and railways. The R-CNN algorithm was applied to automatic detection through the hyperbolic pattern in B-scan images. The algorithm and pre-processing were operated simultaneously along with the GPR to achieve real-time operation. Third, since the incidents of terrorism in Thailand’s southern area, which are associated with the characteristics of buried IEDs, are unique, experimentation with practical scenarios of GPR operations is therefore necessary. The essential parameters of the pre-processing of the GPR detection system were investigated to find their appropriate values, particularly for the application of the real-time automatic detection of IEDs buried under roads and railways.

## 2. Proposed Ground-Penetrating Radar

**Figure 1:**
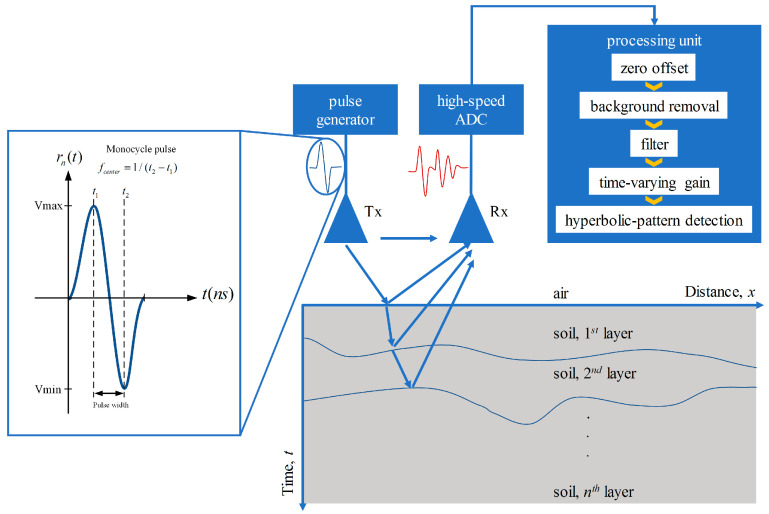
Basic concept and configuration of the GPR system along with signal processing.

The GPR measures and records EM reflection caused by the different media layers in order to find and detect buried objects. Figure 1 depicts the basic concept of the GPR system, consisting of a pulse generator, a processing unit, and transmitting and receiving antennas. A short EM pulse is generated by using a pulse generator and then transmitted through a transmitting antenna. In this paper, the monocycle EM pulse is chosen as seen in the figure. The transmitted pulse is propagated through the ground and reflected in turn to a receiving antenna when different medium layers exist. The received backscattering pulse is collected and then formed as A-scan signals and B-scan images. The processing, including peak detection, zero offset removal, background removal, filtering, time-varying gain, and hyperbolic-pattern detection, is performed within a processing unit [41].

### 2.1. Pre-Processing

Pre-processing, including zero offset removal, background removal, filtering, and time-varying gain, is required before performing the automatic detection of the hyperbolic pattern associated with a buried IED. The backscattered signal, often referred to as an A-scan signal received by a receiving antenna, can be noted as *r*(*t*). The received A-scan signal generally comprises alternating current (AC) and direct current (DC) components. The DC component is caused by many factors, such as ADC, and DC leakage current, and needs to be eliminated first. The zero offset removal is therefore introduced in order to ensure that the mean value of the received A-scan signal is approximately zero. The amplitude probability distribution of the received A-scan signal is assumed to be symmetric around the mean value and not oblique. Additionally, the mean value in the short term is constant over the time duration of the received A-scan signal. The equation of the zero offset removal can be expressed as

(1) $${r^{\prime }}_{n}(t)=r_{n}(t)-\frac{1}{N}\sum_{n}^{N}r_{n}(t)$$

where *r_n_*(*t*) and *r′_n_*(*t*) denote the A-scan signals at the nth sample before and after performing zero offset removal, respectively. *N* is the total sample of the A-scan signal. Background removal is essential for removing the effect caused by environments, especially ground bouncing and the mutual coupling of antennas. In the procedure of the background removal, an A-scan signal collected using the GPR system operated in a normal environment without buried objects is first required, noted as *r_f_*(*t*). The signal is subtracted from that obtained from the zero offset removal as given by

(2) $$x(t)=r^{\prime }(t)-r_{f}(t)$$

where *x*(*t*) denotes the A-scan signal obtained from the background removal. Bandpass filtering is used to eliminate the noise and unwanted signals caused by clutter. The bandpass filtering can be expressed as

(3) $$x^{\prime }(t)=F(x(t))$$

where *x′*(*t*) denotes the A-scan signal obtained after bandpass filtering. Finally, time-varying gain is optional or is required to compensate for the A-scan signals whose amplitude is reduced along the time axis corresponding to the depth, because of propagation path losses as a function of time. In this paper, linear time-varying gain is used and given by

(4) $$y_{n}(t)={x^{\prime }}_{n}(t)kn$$

where *x′_n_*(*t*) and *y_n_*(*t*) denote A-scan signals at the nth sample before and after the processing of the time-varying gain. The *k* is a gain factor [42].

### 2.2. Automatic Detection Using R-CNN

**Figure 2:**
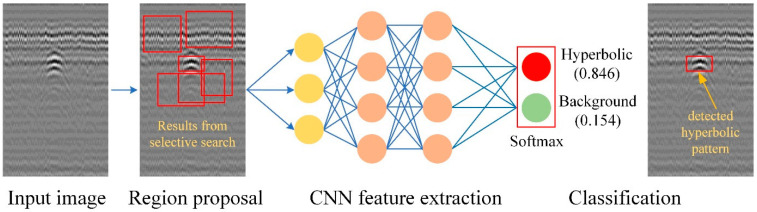
R-CNN object detection systems overview.

The R-CNN was first proposed in [30] by slightly modifying the conventional CNN with the introduction of region proposals exploited to localize the objects within an image. Figure 2 depicts the basic architecture of the R-CNN consisting of three main procedures as follows.

1. Region proposals: The input image is fed to extract the region proposals or regions of interest. A method to search for the object of interest in the whole image is required in order to localize the object within an image.
2. Feature extraction: A fixed-length feature is extracted from the region proposed by using a CNN consisting of three main types of layers to build CNN architecture, namely a convolutional layer, a pooling layer, and fully connected layers.
3. Region classification: The output of the CNN is the scores or weights generated by the SoftMax layer. The scores or weights indicate the class of the objects and the location of the regions within the image. Nonmaximal suppression (NMS) is then used to eliminate overlapping bounding boxes based on their scores and to find the maximum score from the scores in order to identify the object.

## 3. Hardware and Software Implementations

In this paper, the hardware and software implementation of the GPR system is proposed. The proposed GPR system can be used to detect the hyperbolic pattern associated with IEDs buried under roads and railways in real time. In this section, the hardware and software implementation of the proposed GPR system is discussed.

### 3.1. Hardware Implementations

**Figure 3:**
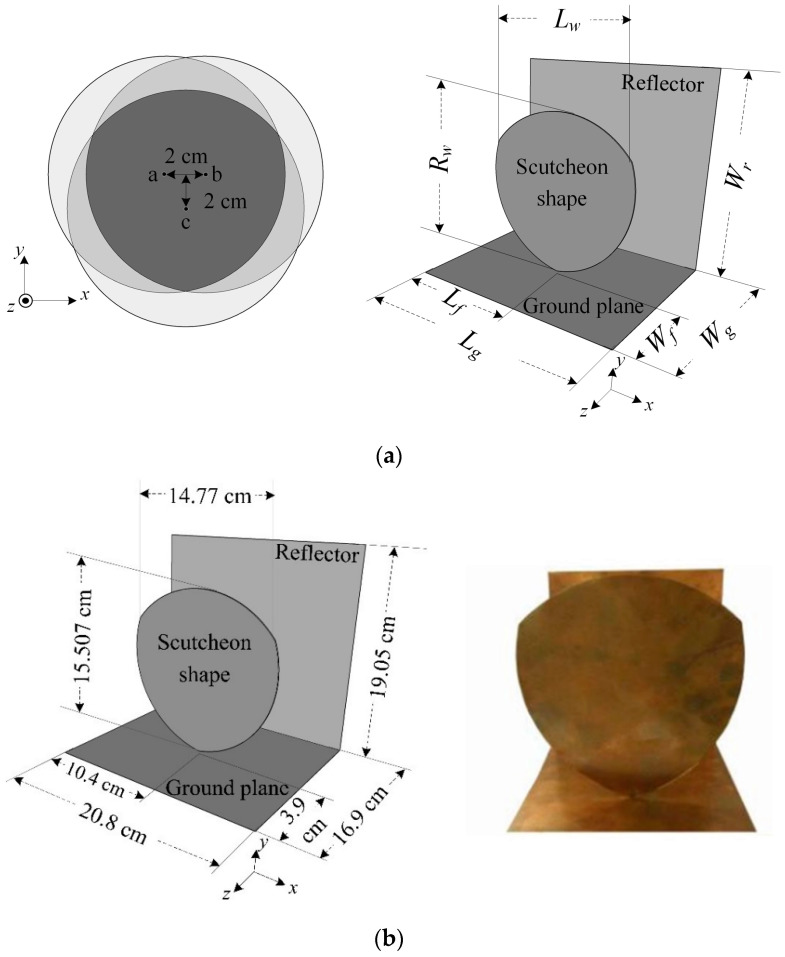
The scutcheon antenna: (**a**) geometry and parameters; (**b**) dimensions and photograph.

One of the important components of the GPR system is the antenna. In this paper, the scutcheon antenna is proposed as the transmitting and receiving antennas of the GPR system. Figure 3a shows the geometry and parameters of the proposed antenna. The proposed antenna consists of three parts of the conductor, namely the scutcheon shape, the finite-size ground plane, and the reflector. The substrate of the antenna is chosen as air in order to achieve high gain, wide bandwidth, and low cost. The scutcheon shape, which is fabricated from three circular plates, comprises a three-part intersection, where the main radiator is connected to the feed point via the finite-size ground plane.

The scutcheon shape with a length (*Lw*) of 14.77 cm and a width (*Rw*) of 15.507 cm is perpendicular to the finite-size ground plane whose length (*Lg*) and width (*Wg*) are 20.8 cm and 16.9 cm, respectively. The three-part intersection of the scutcheon shape consists of the circle plate (radius 7 cm) conductor made from “a” circle plate, “b” circle plate, and “c” circle plate, as seen in Figure 3a. The a, b, and c circle plates are used to form an isosceles triangle as a radiator with a 2-cm base (a-b) and 2-cm height (c⊥ab). The scutcheon shape, which is parallel to the reflector with the dimensions of *Lf* = 20.8 cm and *Wf* = 16.9 cm), will resonate when its length is approximately *λ*/4, where *λ* is the wavelength at the designed lower frequency of 500 MHz. Three corners and the appropriate distance of the intersection shape produce the wider bandwidth of the antenna.

The CST Microwave Studio EM simulator was used to optimize the antenna parameters [36]. Figure 3b shows the dimensions and a photograph of the prototype antenna fabricated from a 1-mm-thick copper plate. A 50-Ω SMA connector is used as a feeding point, which is 1 mm away from the radiator and the ground plane. The parasitic patch is bolted between the radiator and the reflector with plastic glue with spacing in the equilibrium configuration. The SMA connector’s outer conductor is bolted to the ground plane, while its inner conductor is connected to the radiator. It can be seen that the structure of the proposed antenna is simple and easy to assemble.

**Figure 4:**
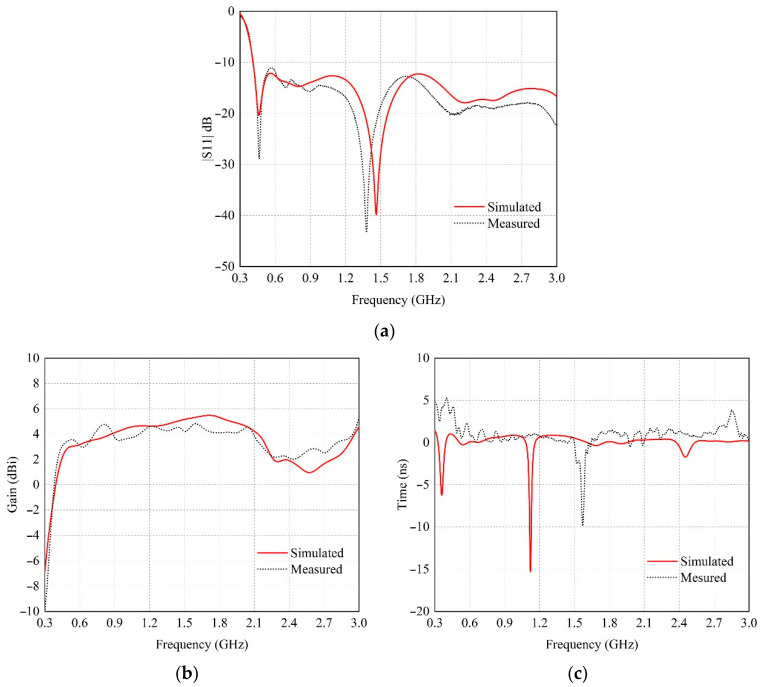
The simulated and measured: (**a**) |*S*11|; (**b**) gain; (**c**) group delay.

The prototype antenna was measured in an anechoic chamber using the far-field measurement system and a Rohde and Schwarz ZNLE6 network analyzer. Figure 4a shows the measured and simulated amplitude of the scattering coefficient |*S*_11_| of the proposed antenna, which is relative to frequency. The measured result showed good agreement with the simulated one. The measured |*S*_11_|, which is lower than −10 dB, covers a frequency range of 0.4 GHz to 3.0 GHz (217% bandwidth), which encompasses the entire UWB GPR frequency band. Figure 4b shows the measured and simulated gain relative to frequency at the maximum direction of the proposed prototype antenna. The measured gain is higher than 2 dBi over the operating UWB GPR frequency band of 0.4 to 3.0 GHz, with the maximum gain of 4.8 dBi at 1.6 GHz. Moreover |*S*_11_| and gain, the group delay relative to the frequency of the proposed antenna, should be determined as illustrated in Figure 4c. The delay group of the antenna is made up of contributions from two components, i.e., the input impedance and the far-field phase. In the figure, the group delays obtained from the simulation and measurement are almost identical and flat throughout the operating frequency band from 0.4 GHz to 3.0 GHz, and up to −12.2 ns at 1.57 GHz for measurement.

**Figure 5:**
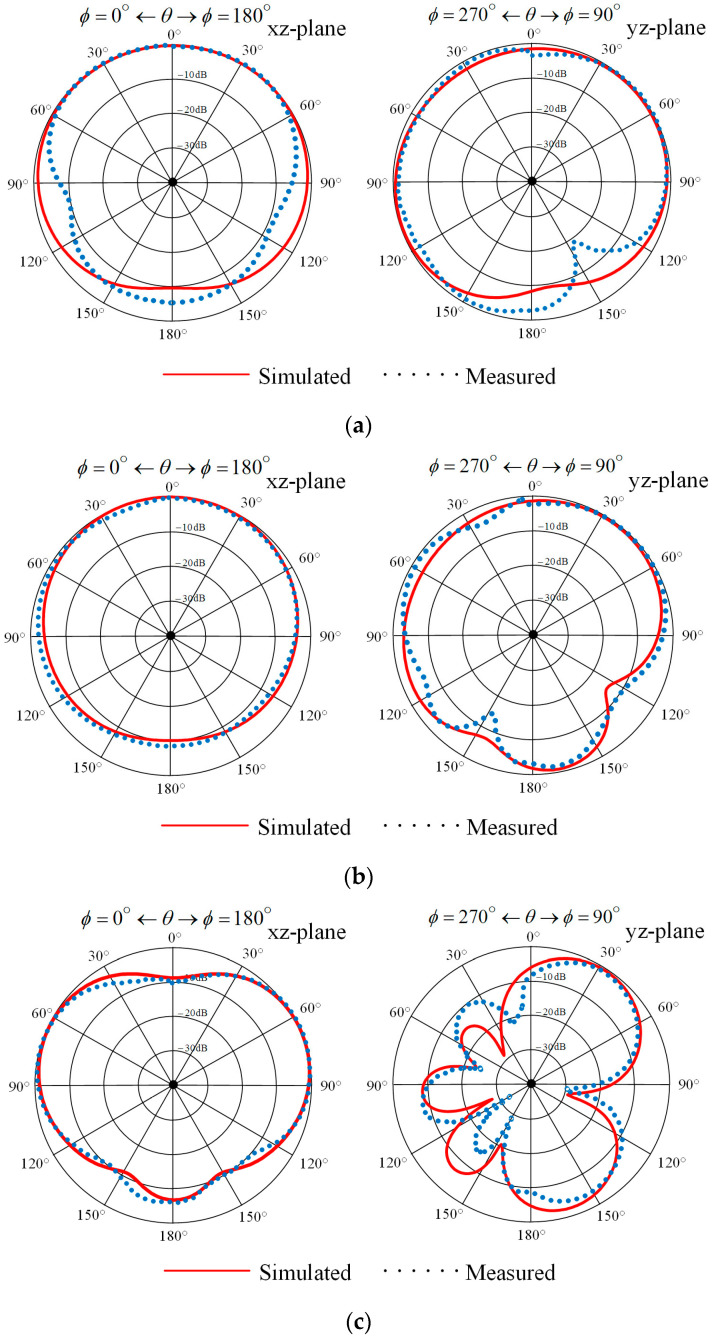
The simulated and measured radiation patterns of the proposed antenna in the *xz* and *yz* planes at: (**a**) 0.4 GHz; (**b**) 0.9 GHz; (**c**) 1.5 GHz.

Figure 5a–c show the measured and simulated radiation patterns in the *xz* and *yz* planes at the operating frequency of 0.4, 0.9, and 1.5 GHz, respectively. In both planes, the radiation patterns are almost unidirectional. The patterns are slightly tilted due to the presence of the ground plane and the reflector, which results in maximum-power generation in the 60° direction at the operating frequency higher than 0.9 GHz. However, the antenna gain at the main direction is degenerated from that at the maximum-power direction. In practical terms, the antenna should be installed so as to be tilted at 30° in order to point the main beam in the desirable direction. According to the figure, the simulated and measured front-to-back (F/B) ratios of the prototype antenna were greater than 3 dB for the operating frequencies of 0.4 GHz, 0.9 GHz, and 1.5 GHz. This implies that the unidirectional antenna pattern was achieved. From the measurement, the half-power beamwidths (HPBWs) of the prototype antenna were 260°, 196°, and 73.6° for the *xz* planes at the operating frequencies of 0.4 GHz, 0.9 GHz, and 1.5 GHz, respectively. For the *yz* planes, the HPBWs were 280°, 81°, and 44° for the operating frequencies of 0.4 GHz, 0.9 GHz, and 1.5 GHz, respectively.

In addition, Table 1 summarizes the characteristic comparisons of the proposed antenna with others published in [36,37,38,39,40]. The operating frequency bands, gains, mechanisms, and sizes were compared. According to the table, our proposed antenna achieves a wider operating frequency, especially at low operating frequency, which can penetrate the ground well. The proposed antenna still maintains a compact size when compared with others.

The scutcheon antenna design is based on broadband impedance matching. There are many antenna shapes used in GPR such as bowtie antenna, horn antenna, spiral antenna, and others. They are widely used in GPR systems due to their wide bandwidth configurations. By comparing the proposed antenna with others at the same lower frequency, the size of the proposed antenna is suitable because the overall antenna shape is cuboid. Moreover, the spiral antenna at the same frequency has a wider cross-sectional area than the proposed antenna.

In terms of performance, the proposed scutcheon antenna has better impedance matching characteristics compared to previously existing antennas. Matching impedance of the scutcheon antenna retains a reflection coefficient of less than −10 dB at frequencies greater than 3 GHz. Compared to the horn and bowtie antennas, the bandwidth is narrower than the proposed scutcheon antennas. However, the pattern of the scutcheon antenna exhibits some flaws because the main beam angle is changed. The horn antenna provides a better boresight radiation pattern throughout the operating frequency. Compared to the bowtie antenna and spiral antenna, these antennas have bidirectional patterns, whereas the application of interest requires additional reflectors to achieve a unidirectional pattern. In terms of gain, the scutcheon antenna possesses a similar gain at the low frequencies of the other antennas. From the point of view of antenna installation, the scutcheon antenna has a ground plane connected to the reflectors. These two sides can be mounted to the metal frame in the GPR system without affecting the impedance matching. 

Generally, the GPR system that operates at the lower frequency range can penetrate through the ground deeper than that of the higher frequency range. However, its resolution of detection will be low because of the narrower bandwidth. In our application of the detection of IEDs buried under the road and train, the center frequency of 800 MHz is suitable for this application, a few centimeters depth of a buried IED, and the soil type of Thailand.

**Figure 6:**
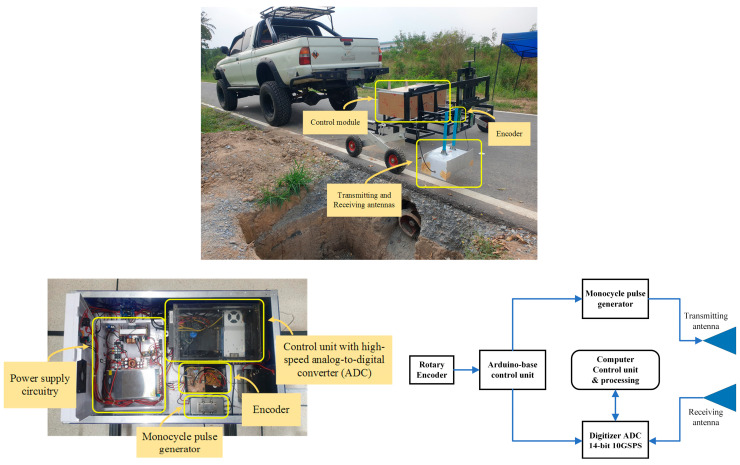
Implemented hardware for GPR.

**Figure 7:**
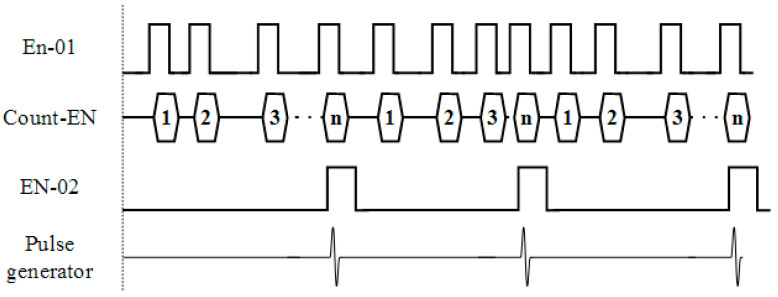
Timing diagram of the control unit.

The proposed GPR system, whose hardware consists of a monocycle pulse generator, a high-speed ADC, rotary encoder distance measurement, power supply circuitry, and a control unit, was implemented as shown in Figure 6. A short monocycle pulse with the center frequency of 800 MHz was generated by using an AVI-V-B pulse generator from the Avtech module. The amplitude and width of the pulse were 50 Vpp with a 50-ohm load, and a pulse repetitive frequency greater than 200 kHz, respectively. The backscattered signal received by the receiving antenna was digitized by using a 14-bit ADC of an ADQ7DC Teledyne SP device and then fed to the computer-based processing unit. The sampling frequency of the ADC was 10 GSPS. The pulse generator and ADC were controlled by the programed Arduino-based control unit with the operating clock frequency of 16 MHz. The frequency of the acquired data fed to the processing unit is programmable depending upon the distance interval of each measurement, which can be arbitrarily set. A rotary encoder was installed on the wheel of the GPR system in order to measure the distance. Figure 7 is a timing diagram of the control signals of the GPR system. A low-rate rectangular pulse was created from the rotary encoder. The pulse logic (EN-01) was changed from “0” to “1” when the distance measured by the rotary encoder reaches the set value corresponding to the measurement resolution, designed as 5 mm. The control unit received and counted the pulse. The control unit sends the trigger signal (EN-02) to the pulse generator in order to generate a short monocycle pulse and simultaneously sends it to the ADC in order to capture the GPR data when the number of the counted pulses (Count-EN) reaches that corresponding to the distance interval of each measurement.

### 3.2. Software Implementations

**Figure 8:**
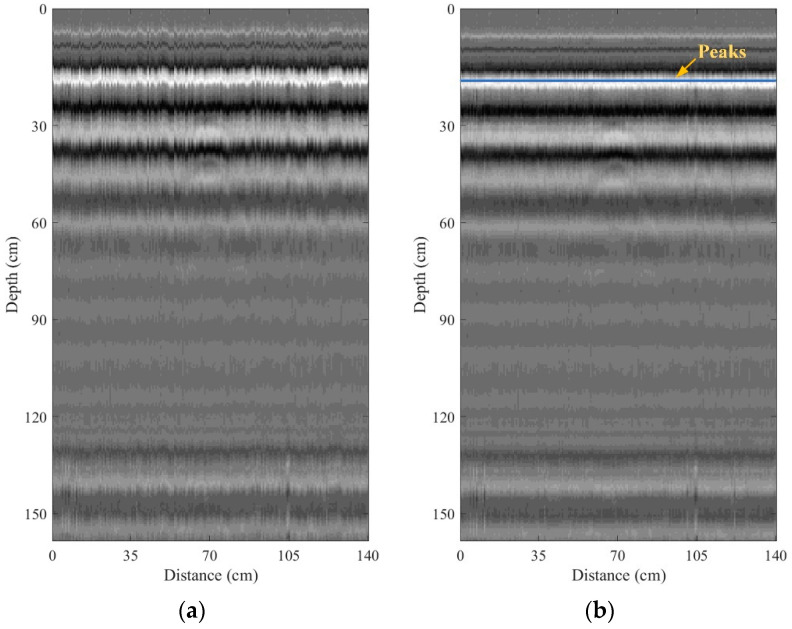
Examples of the B-scan image: (**a**) before; (**b**) after performing the time alignment.

The short EM pulse was transmitted and then received via the transmitting and receiving antennas, respectively. The received A-scan signal was continuously digitized by using the high-speed ADC. The number of samples of the digitized A-scan signal can be adjustable. If the amplitude of the digitized A-scan signal is higher than the threshold predefined by the software of ADC, the signal will be stored in the RAM of the ADC. This is referred to as the level trigger-based capturing method. The stored A-scan signal was automatically fetched from the implemented software and then stored in the RAM of the computer-based processing unit via a PCIe port. Since the transmitting and receiving pulse cannot be perfectly synchronized, the peak of the individual stored A-scan signal was detected by the implemented software. The peaks of the A-scan signals were then aligned at the same time. The aligned A-scan signals were employed in order to form a B-scan image. Figure 8 shows examples of the B-scan image before and after performing the time alignment. In the figure, the B-scan image obtained before performing the time alignment is disorganized because the A-scan signal cannot be captured at the same time. This can be resolved by detecting and aligning their peaks, as seen in the results in Figure 8b.

**Figure 9:**
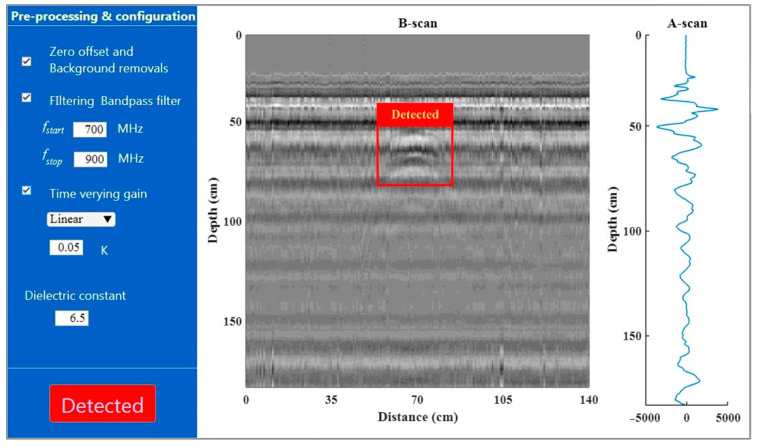
Software graphic user interface.

The computer-based processing unit received the GPR data acquired from the ADC. The software for performing the signal processing of the proposed GPR system was implemented on the Windows operating system. The graphic user interface of the software was designed as shown in Figure 9, where the A-scan signal and the B-scan image were plotted. In the software, the configuration can be divided into two main parts, namely selecting the pre-processing procedures, and setting the parameters. In the first one, pre-processing such as filtering, background removal, and/or time-varying gain can be chosen to be performed in the software in order to enhance the hyperbolic pattern in the B-scan images before automatic detection. In the second one, the parameters for forming the B-scan images and pre-processing can be set. For example, the dielectric constant of the ground was adjustable, resulting in depth in the B-scan images. An A-scan signal is filtered by using a bandpass digital filter in order to eliminate unwanted signals caused by clutter or disturbing noise. The range of the passed frequency of the filter was adjustable as well. The weighting factor of the time-varying gain can be chosen.

**Figure 10:**
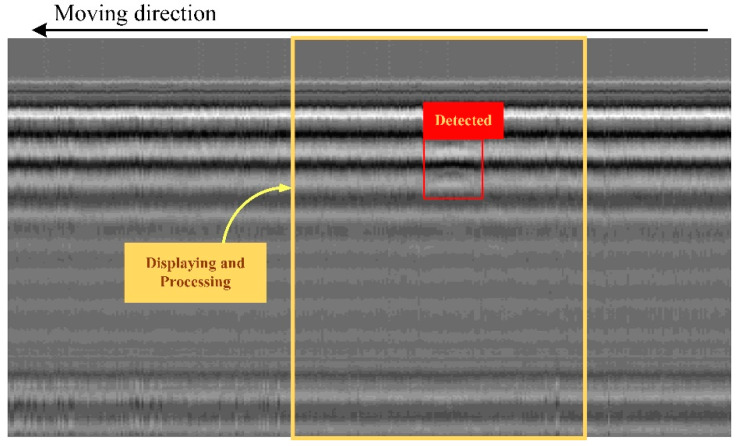
Shift procedure of the B-scan image.

The automatic detection for the hyperbolic patterns in the B-scan image must be included in the software. The A-scan signals collected by using the GPR system and performed by using the pre-processing procedures were stored, simultaneously displayed, and then jointed with each other in a moving direction as a B-scan image. In real-time detection, the B-scan image displayed in the software was framed as an example, as seen in Figure 10. The framed B-scan image was fed to the R-CNN-based detection for the hyperbolic pattern. A new A-scan collected from the GPR and obtained after performing pre-processing will be inserted into the last trace of the B-scan image while the B-scan image will be shifted out. This operates on a first-in, first-out (FIFO) principle. The newly framed B-scan image will be fed to the detection process again.

In addition, Table 2 summarizes the characteristic comparisons of our proposed GPR system with others published in [12,19,24,43]. Note that the GPR system proposed in this paper was implemented along with the R-CNN-based detection, particularly for IEDs buried under roads and railways. This implies that the scenario of the insurgency is unique, therefore requiring a tailor-made GPR system.

## 4. Experimental Setup

**Figure 11:**
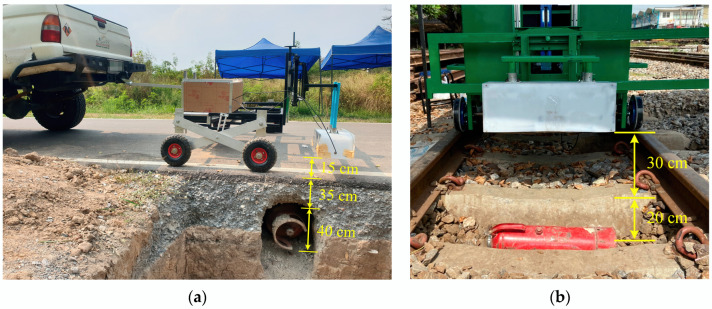
Experimental setup of the implemented GPR for detection of an IED buried under: (**a**) a road; (**b**) a railway.

In this section, the experiments conducted in order to verify the detection performance of the proposed and implemented GPR are discussed. The experimental setup of the proposed GPR is shown in Figure 11. The experiments are divided into two main categories, that is, different experimental situations. First, the operation of the pickup truck-mounted GPR was conducted along a road constructed by three layers, i.e., a subbase, base, and surface courses made of compacted soil, compacted gravel, and asphalt, respectively, as shown in Figure 11a. A hole with a diameter of 40 cm was excavated beside the road. The depth of the hole, measured from the surface of the top road layer, was 35 cm. A gas tank normally packed with the IED and then deployed as a landmine was installed inside the hole. This situation has often occurred because of Thailand’s southern insurgencies [1,2]. In order to collect the data, the GPR mounted on the pickup truck moved at the speed of about 40 km/h. The distance interval of each measurement of the GPR was set equal to 5 mm. As well as insurgency attacks along roads, terrorist attacks have also taken place in public train transportation as well. Thus, in the second experimental scenario, GPR mounted on a maintenance train was operated along the railway, which was paved with large rocks, as shown in Figure 11b. The gas tank representing the IED was buried under the railway. In the experiments, the position of the buried IED was in the middle of the railway, as seen in the Figure 11b. The depth of the buried gas tank was 20 cm. The maintenance train carrying the GPR ran at the speed of not more than 30 km/h in order to collect the GPR data. The speed of the maintenance train was limited because of safety policy; however, the speed does not affect the collected GPR data if the distance interval of each measurement of the GPR is set to be constant. It merely involves the interface between the ADC and the processing unit of the GPR system. At each measurement position, an A-scan signal was collected. Many collected A-scan signals were formed as a B-scan image. Concurrently, real-time automatic detection using the R-CNN for the hyperbolic pattern associated with the buried IED was performed.

## 5. Training Process

**Figure 12:**
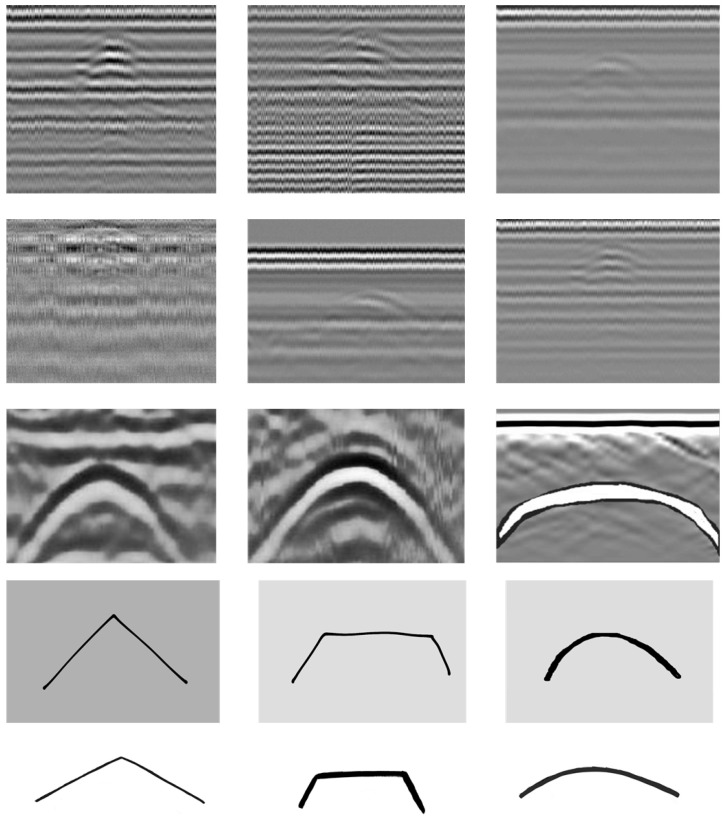

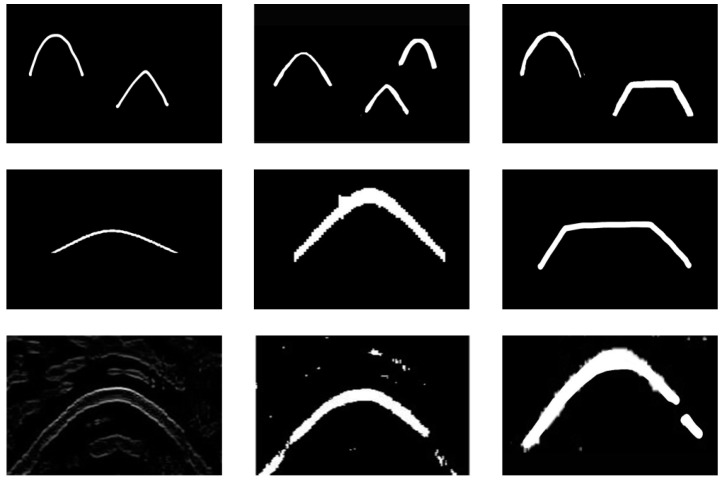
Examples of B-scan images employed as training data.

In order to apply the R-CNN to real-time automatic detection for the hyperbolic pattern associated with the buried IED, a training process is first required. In the R-CNN, the network was designed, comprising one input layer, three convolution layers, and one fully connected layer. For the training process, sixty B-scan images containing the hyperbolic pattern were created using three different methods. The two-third, one-half, and one-six of all B-scan images employed for the training process were obtained from manually drawing, experiments in the laboratory and field, and software simulations, respectively. B-scan images, obtained from experiments in the laboratory where the small road was constructed by many small rocks, were employed for training. In the experiments in the field, there were various scenarios to create B-scan images. Simulations with various conditions were conducted by using the GPRmax software simulator in order to create B-scan images employed for training. Figure 12 illustrates some examples of the B-scan images employed as training data. In the figure, there are various types of B-scan images. The backgrounds of the B-scan images were white, grey, or black, and some of the B-scan images contained one, two, and three hyperbolic patterns. The apexes of the patterns associated with the buried IEDs were sharp, edged, and curved. The hyperbolic patterns were thin and thick. The dimensions of the images were 380 × 350 and 600 × 700 pixels. All of the created B-scan images were then fed to the input layer of the designed network. The feature of the B-scan images was extracted using convolutional layers. In order to decrease the size of the feature maps, a Max-pooling layer was inserted right after the three convolution layers. The feature maps extracted and shrunken by using the convolution layer were fed to fully connected layers.

## 6. Experimental Results

In this section, the experimental results are shown in order to verify the detection performance of the proposed GPR system along with the R-CNN algorithm. They are divided into main categories, namely the proposed GPR system, which surveyed the road and railway, as shown below.

### 6.1. Road

**Figure 13:**
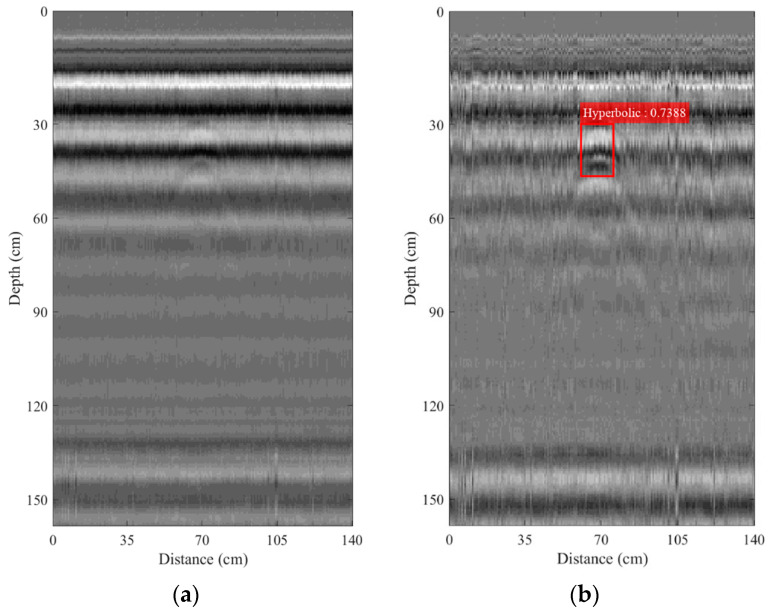
B-scan images collected by using the pickup truck-mounted GPR system on a road: (**a**) without any pre-processing; (**b**) with zero offset and background removals.

**Figure 14:**
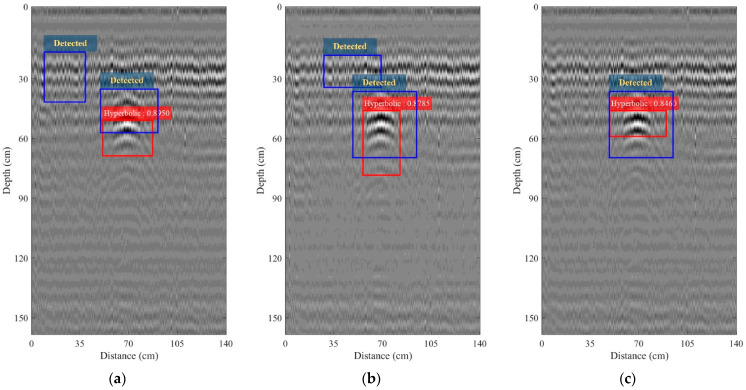
B-scan images collected by using the pickup truck-mounted GPR system on the road and obtained from performing bandpass filtering: (**a**) *Bf* = 100 MHz; (**b**) *Bf* = 150 MHz; (**c**) *Bf* = 200 MHz.

Figure 13a depicts a B-scan image formed from the A-scan signals collected by using the pickup truck-mounted GPR system used to survey the IED buried under the road, as described in Section IV. Clearly, there is no hyperbolic pattern associated with the buried IED in the B-scan image. Furthermore, the ground surface cannot be identified. The R-CNN algorithm was then applied in order to detect the hyperbolic pattern. The technique of region analysis proposed in [44] was applied as well in order to compare its detection performance with the proposed R-CNN algorithm. Figure 13a illustrates that the two detection techniques, R-CNN and region analysis, could not detect the hyperbolic pattern in the B-scan image without any pre-processing. Zero offset and background removals were performed for all collected A-scan signals, which were then formed as a B-scan image, as shown in Figure 13b. In the figure, the small hyperbolic patterns do not appear clearly at the depth of 35 cm in the B-scan image. The detection software using the R-CNN algorithm was utilized in order to detect the hyperbolic pattern in the B-scan image. The red rectangular box, which encompasses the hyperbolic patterns appearing in Figure 13b, indicates that the hyperbolic pattern was detected using the R-CNN algorithm. Following [45], the intersection-over-union (IoU) metric was used to evaluate the accuracy of image detection. It can be given by

(5) $$\mathrm{IoU}=\frac{\left|A\cap B\right|}{\left|A\cup B\right|}\times 100$$

where *A* and *B* denote the areas of the ground-truth bounding box and predicted bounding box, respectively. The ground-truth bounding boxes are the hand-labeled bounding boxes from the B-scan images employed as the training data where the hyperbolic pattern appears. The IoU can be used to determine true positives and false positives in a set of detections. To decide the detection as either true positive or false positive, the threshold of IoU must be chosen. Following [45], the threshold of the IoU was set as 50%. According to the detection in Figure 13b, the IoU was 73.88%. However, the hyperbolic pattern was not detected by using region analysis. The IoU cannot be obtained from the use of region analysis because the difference of scale (DS) is employed as an indicator in order to detect the hyperbolic pattern instead of the use of image recognition [45]. Subsequently, bandpass filtering was performed in order to remove the unwanted signals due to noise and surrounding effects. The center frequency of the filter was fixed at 0.8 GHz corresponding to that of the transmitted monocycle pulse. The bandpass of the filter was varied as *Bf* = 100, 150, and 200 MHz. The B-scan images obtained after performing the bandpass filtering with different bandpasses, *Bf*, are shown in Figure 14. The R-CNN algorithm was utilized again in order to detect the hyperbolic pattern in the B-scan images obtained after performing the bandpass filtering. All of the B-scan images containing the hyperbolic patterns were completely detected, as seen in the figures. The IoUs were 89.49%, 87.85%, and 84.6% when *Bf* = 100, 150, and 200 MHz, respectively. It is clearly seen that the bandpass of *Bf* = 100 MHz achieved the highest IoU and so is suitable for the operation of the R-CNN-based automatic detection of the hyperbolic pattern associated with the buried IED. Additionally, bandpass filtering can significantly increase detection performance. However, when applying region analysis to IED detection instead of R-CNN, there exist mistakes in the detection when *Bf* = 100 and 150, as seen in Figure 14a,b, where the blue rectangular box denotes the detected object.

**Figure 15:**
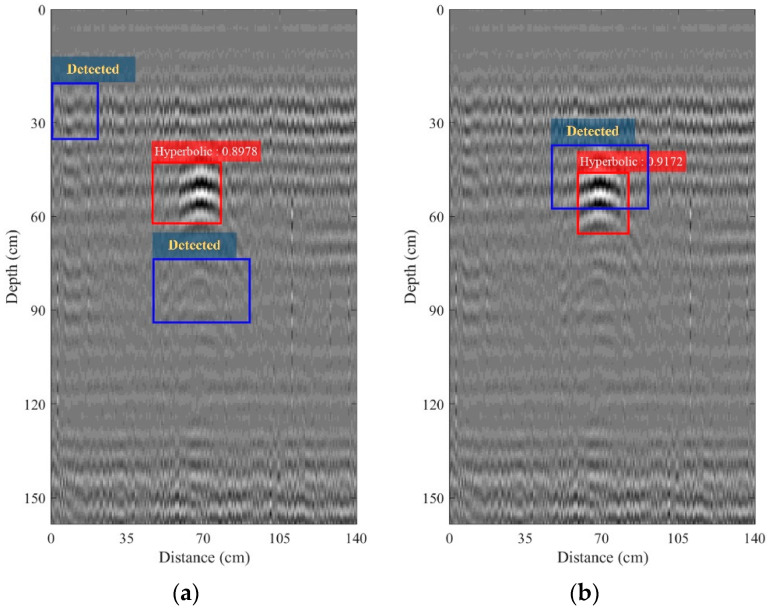
B-scan images collected by using the pickup truck-mounted GPR system on the road and obtained from performing the time-varying gain: (**a**) *k* = 0.05; (**b**) *k* = 0.1.

The B-scan image obtained from performing the bandpass filtering with *B_f_* = 100 MHz was chosen to be amplified along the depth by using the time-varying gain. Figure 15a,b, depict the B-scan images obtained from performing the time-varying gain with a gain factor *k* of 0.05 and 0.1, respectively. The proposed detection software using the R-CNN algorithm will still detect the hyperbolic patterns in the B-scan image as framed by the red rectangular boxes. The IoUs obtained from using the gain factor *k* of 0.05 and 0.1 were 89.78% and 91.72, respectively. On the other hand, the region analysis could detect the hyperbolic pattern correctly, as seen in Figure 15a, where the blue rectangular box encompasses two wrong objects. With the time-varying gain, the layer at many centimeters was significantly more notable. Since the IED was buried at only 35 cm, there was therefore no need for the time-varying gain, which is optional.

### 6.2. Railway

**Figure 16:**
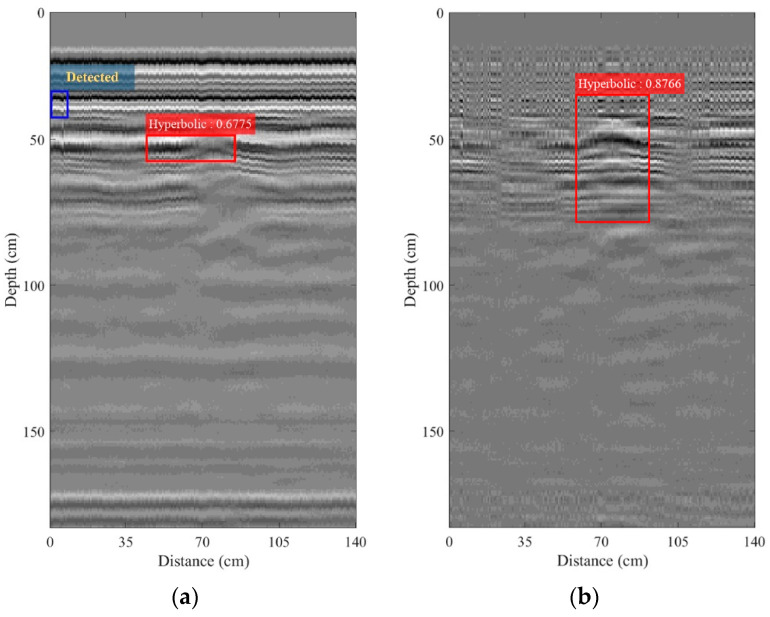
B-scan images collected using the maintenance train-mounted GPR system on the railway: (**a**) without any pre-processing; (**b**) with zero offset and background removals.

The experiments with the GPR mounted on the maintenance train and surveyed on a railway were conducted in order to verify the performance of the proposed GPR system. Figure 16a,b depict the B-scan images collected by the GPR system and performed with and without zero offset and background removals, respectively. As discussed above, the hyperbolic pattern, associated with the buried IED, did not appear in the B-scan image without any pre-processing, while it was unclearly seen in the B-scan image obtained from the zero offset and background removals. However, the R-CNN-based proposed software was able to detect the hyperbolic pattern in the B-scan image without any pre-processing, with the IoU of 67.75%. The IoU of detection of the hyperbolic pattern in the B-scan image obtained from performing zero offset and background removals was 87.66%. It can be noted that the zero offset and background removals can improve detection performance. In contrast, the region analysis still failed to detect the hyperbolic pattern associated with the buried IED.

**Figure 17:**
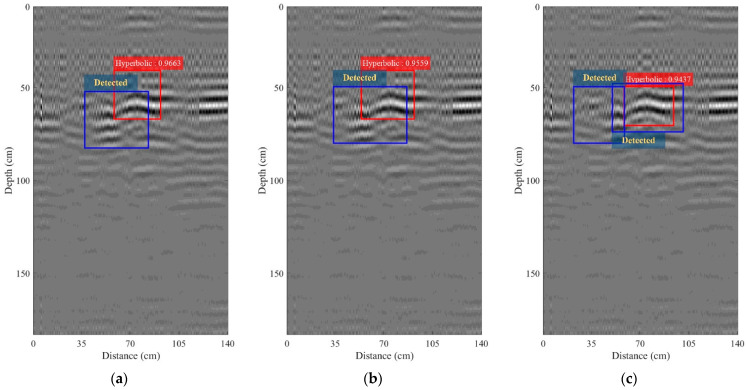
B-scan image collected using the maintenance train-mounted GPR system on the railway and obtained from performing bandpass filtering: (**a**) *Bf* = 100 MHz; (**b**) *Bf* = 150 MHz; (**c**) *Bf* = 200 MHz.

**Figure 18:**
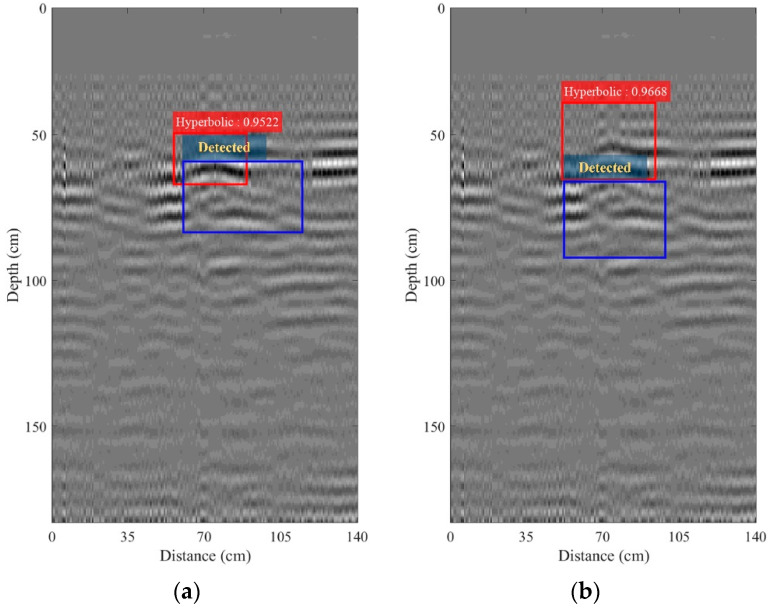
B-scan images collected using the maintenance train-mounted GPR system on the railway and obtained from performing the time-varying gain: (**a**) *k* = 0.05; (**b**) *k* = 0.1.

Figure 17a–c depict the B-scan images obtained after performing bandpass filtering with *B_f_* = 100, 150, and 200 MHz, respectively. Their IoUs were 96.63%, 95.59%, and 94.37%, respectively. Bandpass filtering can significantly improve the R-CNN-based detection of the hyperbolic pattern, and region analysis can detect the hyperbolic patterns, as shown in Figure 17a, while there are mistakes in the detection, as seen in Figure 17b,c. The B-scan image obtained from performing bandpass filtering with *B_f_* = 100 MHz was then fed to the processing of the time-varying gain. Figure 18a,b depict the B-scan images obtained from performing the processing of the time-varying gain with a gain factor *k* of 0.05 and 0.1, respectively. The IoUs achieved 96.68% and 95.22% for *k* = 0.05 and 0.1, respectively.

Table 3 summarizes the performance of the detection using the proposed GPR system. It clearly reveals that the pre-processing assists in increasing the performance of the detection of the IED buried under the road and railway. Note that the proposed GPR system, mounted on the pickup truck and maintenance train, and, respectively, operated on the road and railway, were able to find the buried IEDs very well. This was confirmed by the detection of the hyperbolic pattern in the B-scan images obtained after performing the pre-processing. Finally, it should be noted that if the depth of the buried object is increased, the hyperbolic pattern associated with the buried IED will appear at longer times corresponding to its depth. Moreover, the material of the objects affects the amplitude level of the EM reflections from the objects. The depth, material, and distance do not affect the accuracy of detection.

## 7. Conclusions

In this paper, the implementation of and experimentation with GPR for the real-time automatic detection of buried IEDs are proposed. GPR hardware, consisting of a pulse generator, high-speed ADC, a rotary encoder, and transmitting and receiving antennas, was implemented along with software that included pre-processing and R-CNN in order to achieve real-time automatic detection of the hyperbolic pattern associated with the buried IED. The scutcheon antenna designed particularly for the GPR operation attained an ultra-wideband response. The experiments with the proposed GPR system mounted on the pickup truck and the maintenance train were conducted based on the unique and real situations of Thailand’s insurgency for surveying IEDs buried under roads and railways. The experimental results in terms of the B-scan images collected using the proposed GPR system were shown and they confirmed the good performance of the detection of the hyperbolic patterns in the B-scan images. The IoUs obtained from the detection using the proposed R-CNN-based GPR system with pre-processing were higher than those without the use of pre-processing.

## Figures and Tables

**Table 1:** Characteristic comparisons of the proposed antenna with others.

	|*S*11| (GHz)	Gain (dBi)	Pattern	Mechanism	Size (cm^3^)
[36]	0.25–0.85	4	Unidirectional	Bowtie Antenna	18.0 × 28.2 × 15.0
[37]	0.5–3.0	12	Unidirectional	Bowtie Antenna	18.0 × 27.0 × 4.3
[38]	0.14–0.51	4.9	Unidirectional	Tem Horn Antenna	60.0 × 60.0 × 15.0
[39]	2.0–7.0	-	Unidirectional	Tapered Antenna	28.0 × 0.15 × 43.0
[40]	5.5–10.5	-	Unidirectional	Tapered Antenna	10.1 × 0.15 × 24.8
Proposed antenna	0.4–3.0	4.8	Unidirectional	Monopole Antenna	20.8 × 16.9 × 19.05

**Table 2:** Characteristic comparisons of the proposed GPR systems.

	GPR Type	Classification	Environment	Frequency	Real-Time Detection
[12]	Custom	No	Laboratory	Monocycle Pulse	No
[19]	Commercial	Yes	Field and GprMax	Step frequency	
[24]	Commercial	Yes	Field testing	Step frequency	No
[43]	-	Yes	GprMax	Monocycle Pulse	No
Proposed	Custom	Yes	Field testing	Monocycle Pulse	Yes

**Table 3:** Performance of the detection of the proposed GPR system.

	Without Any Pre-Processing	Zero Offset and Background Removals	Bandpass Filtering (MHz)			Time-Varying Gain (*k*)	
			100	150	200	0.05	0.1
Pickup truck	0%	73.88%	89.50%	87.85%	84.60%	89.78	91.72
Train	67.75%	87.66%	96.63%	95.59%	94.37%	95.22%	96.68%

## Data Availability

Not applicable.
